# Augmented Reality in Interventional Radiology: Transforming Training Paradigms

**DOI:** 10.7759/cureus.54907

**Published:** 2024-02-25

**Authors:** James Baker, Antony Antypas, Prashant Aggarwal, Charlotte Elliott, Robert Baxter, Shwetabh Singh, Naduni Jayasinghe, Daniel Reed, Alexander Boden, Imogen Evans, Bryony Hurst, Andrew Koo

**Affiliations:** 1 Emergency Medicine, Bankstown-Lidcombe Hospital, Sydney, AUS; 2 Orthopedics and Rehabilitation, Leeds Teaching Hospital NHS Trust, Leeds, GBR; 3 Urology, University Hospitals Birmingham NHS Foundation Trust, Birmingham, GBR; 4 Otolaryngology, Stockport NHS Trust, Stockport, GBR; 5 General Practice, Harrogate and District NHS Foundation Trust, Harrogate, GBR; 6 Medical Education, Sandwell and West Birmingham Hospitals NHS Trust, Birmingham, GBR; 7 Emergency Medicine, Bradford Royal Infirmary, Bradford, GBR; 8 School of Medicine, University of East Anglia, Norwich, GBR; 9 School of Medicine, University of Leeds, Leeds, GBR; 10 Internal Medicine, Bradford Royal Infirmary, Bradford, GBR; 11 Radiology, Harrogate and District NHS Foundation Trust, Harrogate, GBR

**Keywords:** medical simulation, augmented reality, medical education, interventional radiology, healthcare tech contest

## Abstract

The ascent of medical technology places augmented reality (AR) at the forefront of potential advancements in interventional radiology (IR) training. This review delves into the symbiotic relationship between AR and conventional IR training, casting light on the opportunities and hurdles intrinsic to this integration. A targeted literature review was conducted using the databases PubMed, Cochrane Library, and Embase. Search terms included (((“Augmented Reality” OR “Virtual Reality”)) AND ((Education OR Training))) AND ((“Interventional Radiology”)). Ten studies identified using the comprehensive inclusion criteria helped scrutinize the use of AR in IR training. Key outcomes include improved procedural accuracy, reduced training duration, and heightened trainee confidence. However, it also identifies limitations such as small sample sizes, reliance on simulation environments, and technological constraints in AR implementation. Despite these challenges, the review underscored the transformative potential of AR in IR education, suggesting its capacity to revolutionize training methodologies. However, it also calls for continued technological development and empirical research to address current challenges and fully leverage AR's capabilities in medical education.

## Introduction and background

Interventional radiology (IR), often described as the convergence of diagnostic imaging and minimally invasive procedures, has firmly established its role in the panorama of modern medicine [1]. IR provides therapeutic solutions, often reducing the need for more invasive surgeries, resulting in shorter recovery times and reduced hospital stays [2]. Traditionally, training in IR has relied heavily on the apprenticeship model, wherein trainees would gain expertise through observing senior clinicians before gradually participating in hands-on practice. Although this approach allows trainees to understand the complexities of live procedures, it may compromise patient safety and increase trainee anxiety [3].

Advancements in technology and a global focus on patient safety have prompted a shift in medical education, especially in fields like IR [4]. The adoption of simulators and other digital tools has offered trainees opportunities to practice in risk-free environments before transitioning to real clinical scenarios [5]. Augmented reality (AR) emerges as a pioneering force in this digital transformation. By superimposing digital information onto the real world, AR offers a unique blend of hands-on and simulated experiences [6]. For IR trainees, who must grapple with the challenges of spatial and cognitive proprioception during training [7], AR offers the ability to visualize complex anatomical structures and guide interventions, enhancing their understanding and refining their skills without compromising patient safety [8]. While numerous studies have delved into the utilization of AR in surgical training, its application in IR remains notably underexplored [9].

There is only one previous literature review on this subject, conducted in 2020. It highlighted the potential of AR use in IR education but expressed some limitations. The review found a lack of diversity in the procedures studied, with only three different procedures being analyzed across the reviewed literature due to the lack of studies at that time. A gap was also identified concerning the study participants; the investigations predominantly focused on novice physicians or non-physicians, side-lining radiologists. Given the limitations in this review and the growing literature in the subsequent years, an updated review is imperative [8]. This literature review aims to provide a snapshot of how AR impacts IR education and to highlight both the transformative potential of AR and areas needing further research.

## Review

Methods

Protocol

A literature review was undertaken following guidelines from PROSPERO and the PRISMA 2020 checklist was used to ensure validity [10]. The literature search for this review was conducted in September 2023. We included papers published up to that point to ensure the most current understanding of the topic was reflected in our review.

Search Strategy and Selection Criteria

A systematic search of the literature was undertaken using established databases: PubMed, Cochrane Library, and Embase. The search strategy was designed to ensure comprehensive coverage, incorporating a combination of relevant terms: “Augmented Reality” OR “Virtual Reality,” “Education” OR “Training,” and “Interventional Radiology.” The Reference lists of identified primary articles were also scrutinized to uncover any potentially overlooked, yet relevant, studies. 

Inclusion and Exclusion Criteria

Eligibility criteria for paper inclusion were based on the PICO framework [11]. *P*: Undergraduate healthcare students, postgraduate trainees, and healthcare professionals undergoing continuous professional development training, irrespective of their specialties. *I*: Use of AR in procedural IR education. *C*: Traditional teaching methodologies versus AR-based instruction. *O*: The outcomes of interest include improvements in ultrasound skills, procedural efficiency, and safety measures, alongside assessing educational engagement, skill retention, and accessibility.

Study Selection 

Eligible studies were identified by two separate, blinded, and independent reviewers (JB, AA), with any disagreements resolved through consensus or, if needed, by consultation with an independent assessor (CE) to ensure an unbiased selection process. Full-text evaluation was undertaken for any studies meeting the selection criteria to ensure eligibility for this targeted literature review. Studies of any publication year were considered, primarily in English. Non-English reports were included if they had an English abstract. The scope encompassed peer-reviewed articles, conference abstracts, and unpublished works. Exclusions were non-empirical papers, articles not focusing on AR in procedural IR education, and opinion pieces without primary data. The review primarily emphasized studies with empirical evidence or insightful qualitative reviews highlighting AR's educational benefits in IR. Studies included in this review were published within the decade from 2013 to 2023, aligning our analysis with the latest developments in AR applications for IR training.

*Data Extraction* 

Upon identifying relevant studies, two reviewers undertook data extraction. Information extracted encompassed study design, methodology, population, sample size, AR technology used, primary outcomes, and limitations. Additionally, participant characteristics, intervention details, and funding sources were gathered. In instances where details were unclear or absent within source documents, we undertook a meticulous approach to assess the potential impact of such gaps on our review's integrity. We differentiated between data presumed non-critical and information crucial but missing, which could significantly influence the review's outcomes. This included systematic documentation of the instances of unclear or absent data, categorizing them based on their relevance to the core objectives of our review. This strategy was aimed at enhancing the transparency and comprehensiveness of our analysis, ensuring a robust understanding of the reviewed studies' context and findings.

Critical Appraisal of Data

The revised Cochrane risk-of-bias tool for randomized trials was applied to assess the risk of bias in individual studies and across studies [12]. We employed the Best Evidence Medical Education (BEME) quality indicators to analyze the selected randomized controlled trials (RCTs), ensuring our review met the high standards of educational research [13].

Results

**Figure 1:**
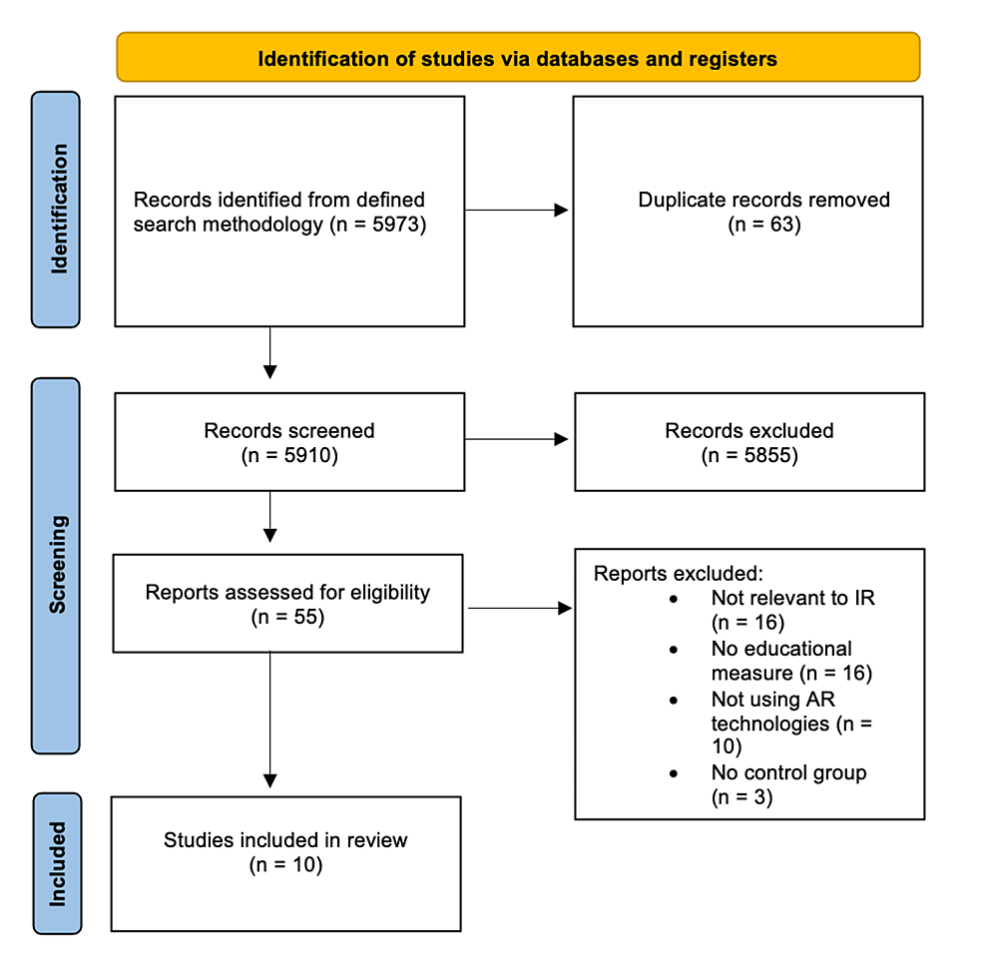
PRISMA flow diagram of study selection PRISMA flowchart depicting the process from the initial identification of 5,973 studies through database searching to the final inclusion of 10 studies in the review, after screening and assessing based on the methodology described, for the relevance to Interventional Radiology (IR) and use of Augmented Reality (AR) technologies.

The search yielded 5,973 articles, with 63 being excluded as duplicates, through analysis of abstracts, 55 were chosen for full-text evaluation, from which 10 were included (Figure 1).

**Figure 2:**
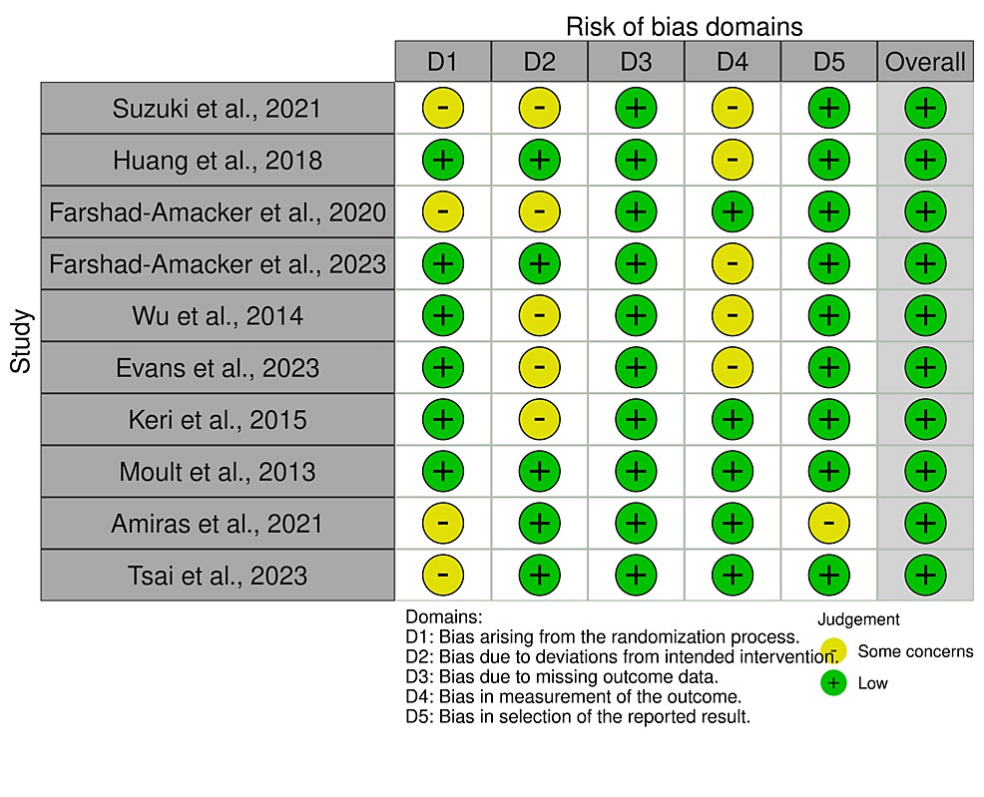
Risk of bias of studies using Cochrane risk-of-bias tool Application of the Revised Cochrane Risk-of-Bias Tool [12]. This tool was utilised to evaluate the bias risk in individual studies as well as collectively across studies included in the analysis [14-23].

The Revised Cochrane Risk-of-Bias Tool was utilized to evaluate the risk of bias in individual studies as well as collectively across studies included in the analysis (Figure 2) [12].

AR technologies, including wearable devices, simulators, and CT simulators, offer distinct advantages for IR education. Wearable devices such as the Microsoft HoloLens™ and Google Glass™ deliver immersive AR experiences by overlaying digital information onto the user's field of view, enhancing spatial understanding and procedural planning. This integration of holographic visuals with the real environment is instrumental in comprehending complex anatomical structures and guiding interventions in real time. The Perk Tutor™ system, representing AR-enhanced simulators, focuses on refining procedural skills like needle insertion by combining ultrasound imaging with real-time 3D visuals. This category underscores skill acquisition in a controlled environment, supporting a broad spectrum of procedural training including ultrasound-guided interventions. Meanwhile, CT simulators provide realistic training environments for CT-guided procedures, with technologies like the Microsoft HoloLens 2™ paired with torso phantoms and AR tracking, enhancing trainees' understanding of CT-guided biopsy techniques and needle placement accuracy.

Wearable AR for Ultrasound-Guided Procedures

Suzuki et al. conducted a study where ten radiologists used a HoloLens™ smart-glass device to simulate cannulation procedures on the internal jugular vein (IJV) and subclavian vein (SCV) using phantom vessels. They trialed three methods: standard US, AR-only, and combined AR-US. Results showed that for SCV procedures, the AR-only and combined AR-US significantly improved performance in subjective evaluation, for both anatomical understanding and self-confidence of the procedure (P < 0.001). On the other hand, there was no statistical difference in subjective evaluation of IJV cannulation for anatomical understanding and self-confidence of the procedure (P = 0.264, 0.071 respectively). An objective analysis of the accuracy of needle puncture showed no significant difference for IJV (P = 0.165), but the AR-only approach showed significant improvement in needle positioning for the SCV (P = 0.034) [14].

Turning our focus to the novice participants. Huang et al. divided 32 novice central line operators into two groups: one utilized AR glasses (Brother AiRScouter WD-200B AR glasses) while the other, the control group, employed traditional US for central venous cannulation (CVC) placement. Before the procedure, all participants were shown a tutorial video. Of these, 71% successfully cannulated the IJV on their first attempt with no statistical difference between the groups. There was a difference in adherence level between the groups, with the AR users scoring 22.9 ± 4.1 and the non-AR users registering 18.1 ± 6.3 (η2 = 0.90; P = 0.003). In a feedback survey conducted among the AR group, over 80% reported that the device did not cause discomfort or fatigue, and stated the displayed information was relevant and promptly responsive. However, 30% mentioned challenges in adjusting the device or felt it impacted their procedural skills. Both the AR and non-AR groups displayed proficiency in IJV cannulation, with the AR group showcasing a higher adherence index. The median total procedure time did not show a significant difference between the two groups [15].

A further study compared US-guided needle placements using conventional methods versus AR US viewing [16]. The system consists of a conventional US machine, with AR displays (Microsoft HoloLens). Three untrained operators (orthopedic surgeons) and two experienced radiologists conducted 200 US-guided punctures. The punctures were performed on a leg phantom containing varied soft tissue lesions using both techniques. Using AR-US resulted in a reduction in puncture time and fewer needle passes. Both techniques had similar accuracy in reaching the correct location: AR-US at 90% and conventional US at 94%. More strikingly, while novices initially took 21.5 s using the traditional method compared to experts' 10.5 s, AR leveled the field, bringing novice times down to 12.5 s against the experts' 13s [16].

Further extending the discourse, Farshad-Amacker et al. conducted a study concentrating on determining the effectiveness of AR US (Microsoft HoloLens TM) versus US for biopsy training on untrained operators. Engaging 44 medical students, each with basic US training but no AR-US exposure, they were tasked with simulating superficial soft tissue biopsies using both modalities. Findings revealed no significant difference in time-to-puncture between AR-US and US. Unsuccessful punctures were minimal (n = 11 for both techniques), with no significant difference between them (p = 0.71). An improvement was observed from the first to the third attempt with AR-US compared to the US. Notably, an overwhelming 79.5% found AR-US to be a more enjoyable and preferable learning method. The research indicates AR's potential in enhancing biopsy training, even if no time efficiency was observed compared to the US [17].

In a study led by Wu et al., the proficiency of 40 participants, comprised of 20 medical students and 20 emergency medicine trainees, in CVC placement was systematically assessed. Before the procedure, participants were oriented with a video tutorial detailing the application of Google Glass and the technique for US-guided placement of an IJV catheter. Participants were then stratified into two cohorts: those employing Google Glass and those who did not. The configuration of the US equipment was standardized across both cohorts. However, the intervention cohort viewed the US images exclusively through the Google Glass interface, whereas the control cohort utilized the conventional US monitor. Notably, participants in the Google Glass cohort experienced prolonged procedure durations and exhibited increased instances of needle repositions, regardless of their educational background. Post-intervention questionnaires highlighted that 75% and 60% of the participants had minimal previous exposure to AR and wearable technology, respectively. However, 73% acknowledged some familiarity with Google Glass, and a significant 87% of Google Glass users reported a comfortable experience during the procedure [18].

AR-Enhanced Simulators for Ultrasound Training

Evans et al. assessed central venous puncture (CVP) performance among 18 foundation training doctors, participants were randomized into two groups: both groups initially received standardized CVC puncture training. One group had a further element of training, incorporating the use of the NeedleTrainer^TM^ for simulation-based training. Both groups underwent a timed assessment involving a CVP on a phantom. The primary outcome, time taken to reach the vein, exhibited no significant difference between the control (median: 50.5 seconds, IQR: 14.0-78.0) and simulation groups (median: 69 seconds, IQR: 34.0-93.5; P = 0.24). Secondary outcomes, including the number of needle passes, observer assessment, and participant confidence, also showed no significant variations between groups. However, the NASA Task Load Index revealed a higher median mental demand in the simulation group (median: 13, IQR: 11.6-14.4) compared to the control group (median: 7.5, IQR: 4.8-9.9; P = 0.002), implying a greater cognitive load during the simulated training process [19].

Keri et al. investigated the Perk Tutor^TM^. 24 anesthesiology and surgical residents were enrolled in a study comparing the Perk Tutor to conventional lumbar puncture (LP) training. Even though the participants varied in their LP and US experience, none had previously used US guidance for LP. Participants were divided into two groups: the AR-US group, which used the Perk Tutor system, and the control group which used the traditional US. To ensure uniform baseline knowledge, training was given using three lumbar spine models with varying degrees of complexity. After the instructional phase, participants' skills were assessed using conventional US on the phantom model alone. Objective performance metrics revealed significant differences between the groups. The AR-US group displayed less potential tissue damage, with a median of 39.7 cm², compared to the control’s 128.3 cm² (P = 0.01). Furthermore, the AR-US group showed a shorter needle path (426.0 mm vs. 629.7 mm; P = 0.02) and needle insertion time (30.3 seconds vs. 59.1 seconds; P = 0.05) than the control group. However, the total procedure time was not significantly different between the groups. The success rate within the ten-minute test duration was 91.6% in the control group and 100% in the AR-US group [20].

Moult et al. delved further into the potential of the Perk Tutor, in improving the performance of novices undertaking a facet joint injection. Twenty-six pre-medical undergraduate students, all inexperienced in needle insertion, participated in the study. Following an initial 10-minute foundational class - covering anatomy, procedure, US interpretation, and needle handling - participants were split into two groups. One group practiced with both the Perk Tutor and a phantom model, while the other had only the phantom model and the US. After practice, both groups were asked to undertake a US-guided facet joint injection. Notably, the AR-assisted educational group showcased a mean success rate of 61.5%, which was significantly higher than the phantom-only group’s 38.5% (P = 0.031). Furthermore, there were no significant differences in procedure times or needle path lengths between the two groups [21].

AR for CT-Guided Intervention Training

Amiras et al. utilized the Microsoft HoloLens 2™ for computed tomography (CT) guided biopsy training. The research team created a simulated environment using torso phantoms with ChArUco markers for AR tracking. This environment was used to practice biopsies on two retroperitoneal lymph nodes of differing complexities. The study involved 16 participants, including 12 junior radiology trainees and four trainers, who used a HoloLens application designed to deliver an interactive and realistic training experience. Feedback collected post-simulation showed that 68.8% of participants found the simulation realistic. The same proportion agreed that the accuracy of the needle placement was satisfactory for the tasks. The acceptability of the training method was high, with 81.3% of users enjoying the experience. About a quarter of the users found the application user-friendly, half were neutral, and a minority found it somewhat difficult to use. The HoloLens hardware was comfortable for most, with 87.5% reporting no discomfort. The training notably improved confidence, with three-quarters of the users feeling more skilled afterward. A significant 81.25% believed the AR simulation to be a valuable complement to their existing training regimen. Among the subgroup of radiology 'experts', 75% perceived the procedure as realistic [22].

Tsai et al. sought to assess the efficacy of postgraduate education in pulmonary nodule computed tomography-guided localization (PNCL) using a phantom simulation, both with and without the assistance of the laser angle guided assembly (LAGA), an innovative angle reference device. Conducted in an academic hospital setting in Taiwan, the study involved a cohort of seven thoracic surgery residents and three senior physicians. These participants engaged in PNCL procedures using a phantom simulation on five different nodules, in scenarios with and without LAGA, and their performance was subsequently evaluated through a questionnaire. The results indicated a notable increase in the confidence level of the participants, with the median score escalating from 7 to 8 on a 9-point Likert scale after the training session (p = 0.001). This improvement in confidence was paralleled by high scores in both enhanced PNCL ability and course satisfaction, 8 (with a range of 5-9) and 9 (with a range of 7-9), respectively. Significantly, the use of LAGA during the procedures was found to enable broader puncture angles, reduce the frequency of punctures, and minimize the deviation in angles. These procedural improvements were quantitatively significant, with LAGA use leading to broader puncture angles of 27.5° (ranging from 0° to 80°), compared to 14° (ranging from 0° to 80°) without LAGA (p = 0.003). Furthermore, the study reported a decrease in puncture frequency (with LAGA: 1 (range 1-4); without LAGA: 2 (range 1-5), p < 0.001) and a reduction in angle deviation (with LAGA: 3° (range 0°-8°); without LAGA: 5° (range 0°-19°), p = 0.002). Additional insights from the study revealed that the experience level of the physicians influenced the efficiency of the PNCL procedures. Notably, physicians with prior PNCL experience performed the procedure more efficiently, and the direction of the needle traversing towards the mediastinum was found to impact both the frequency and duration of the procedure. Despite these advancements, the study interestingly noted that the learning curve did not significantly improve the procedure performance after ten rounds of PNCL simulation [23].

All results are surmised in Table 1. The evident transformative capacities of AR in IR necessitate a judicious scrutiny of its challenges.

**Table 1:** Summary of findings This table presents a comparative overview of studies evaluating augmented reality (AR) in interventional radiology (IR) training. It details the specific procedure being assessed, the type of AR intervention used, control conditions, participant demographics, study design, and key results. US: ultrasound, IJV: internal jugular vein, SCV: subclavian vein, RCT: randomized controlled trial, LP: lumbar puncture, PNCL: percutaneous needle placement, CT: computed tomography, LAGA: localization and guidance accessory.

Study	Procedure	Intervention	Control	Participants	Study Design	Results
Wearable Augmented Reality for Ultrasound-Guided Procedures						
Suzuki et al., 2021 [14]	US vs. AR-US guided IJV + SCV cannulation on a phantom	Microsoft HoloLens^TM^	US only	10 Radiologists	RCT with pre and post exposure questionnaire	There was no subjective difference in IJV guiding methods. AR approach showed significant improvements in needle positioning
Huang et al., 2018 [15]	US vs. AR-US guided IJV cannulation on a phantom	Brother AiRScouter WD-200B AR glasses^TM^	US only	32 Novice Central line operators	RCT with pre and post exposure questionnaire	Required fewer attempts to be successful when using AR training. 30% found difficult to control. 94% indicated that the device’s interactions were straightforward. AR-US educational methods showcased a higher adherence index
Farshad-Amacker et al., 2020 [16]	US vs. AR-US guided needle placement in leg phantoms (soft tissue lesion; vessel phantom)	Microsoft HoloLens^TM^	US only	3 untrained operators (orthopaedic surgeons with no prior US experience) + 2 experienced radiologists	RCT with pre-experience questionnaire	AR-US reduced procedure time and number of needle passes compared with US. Initial gap in performance of untrained versus experienced operators with the conventional US reduced when using AR education.
Farshad-Amacker et al., 2023 [17]	US vs. AR-US biopsy of superficial soft tissue lesions	Microsoft HoloLens^TM^	US only	44 US operators (medical students with no prior biopsy experience but had completed a standard US course)	RCT with pre and post exposure questionnaire	AR-US was considered by 79.5% of the operators to be the more enjoyable means of learning and performing US-guided biopsies. Time to puncture did not differ significantly between AR-US and US.
Wu et al., 2014 [18]	US vs AR-US guided IJV insertion on a phantom	Google Glass^TM^	US only	20 medical students (10 1sy years, 10 4^th^ years) 20 emergency medicine residents (10 1^st^ years, 10 3^rd^ years)	RCT with pre and post-experience questionnaire	Google Glass wearers took longer to perform the procedure at all training levels and required more needle redirections when using AR education.
Augmented Reality-Enhanced Simulators for Ultrasound Training						
Evans et al., 2023 [19]	Traditional teaching method vs AR-enhanced education for US guided IJV needle insertion in a phantom	NeedleTrainer^TM^	US only	18 foundation trainees	RCT with pre and post-experience questionnaire	No significant difference in time taken to reach the vein between the two groups. Higher median mental demand in the simulation group.
Keri et al., 2015 [20]	AR enhanced education vs traditional methods for US guided LP on a phantom	Perk Tutor^TM^	US only	24 anaesthetic and surgery trainees	RCT with pre-experience questionnaire	Less tissue damage and shorter needle path inside tissue when trained using the AR method. No significant difference in overall success rates between methods.
Moult et al., 2013 [21]	Traditional teaching method vs, AR enhanced education for US guided LP on a phantom facet joint injection	Perk tutor^TM^	US only	26 premedical undergraduate students with no prior needle insertion experience	RCT with pre-experience questionnaire	The success rate of the Perk Tutor trained group was significantly higher than the success rate of the Control group.
Augmented Reality for CT-Guided Intervention Training						
Amiras et al., 2021 [22]	CT-guided biopsy training of simulated retroperitoneal lymph nodes	Microsoft HoloLens 2™ paired with torso phantoms and ChArUco markers for AR tracking	Experience of traditional training methods without AR	6 individuals (12 junior radiology trainees and 4 trainers)	Interventional study using simulated environment and feedback questionnaires	68.75% of participants reported the procedure simulation was realistic. 81.25% enjoyed the AR training experience. 75% felt more adept after training. 81.25% endorsed the AR simulation as beneficial for training.
Tsai et al., 2023 [23]	PNCL using Phantom Simulation	LAGA	Standard PNCL without LAGA	7 thoracic surgery residents and 3 experienced senior physicians	Comparative study with a pre-post design	Improvement in confidence level from median post-training. Enhanced PNCL ability and course satisfaction scored. Use of LAGA enabled broader puncture angles, lower puncture frequency, and smaller angle deviation.

Discussion

Our review aims to scrutinize the existing evidence surrounding the utilization of AR in procedural IR training. We endeavored to assess AR’s efficacy in enhancing performance, skill acquisition, and its benefits and challenges throughout the educational process.

The review revealed that AR could offer educational benefits to IR training in various contexts. Suzuki et al. and Farshad-Amacker et al. demonstrated AR's role in improving needle positioning and reducing procedure times. However, it's important to note that the objective assessment of the procedures used a non-standardized scoring tool with uncertain validity. Moult et al. highlighted AR's advantages in training, leading to fewer cannulation attempts and higher success rates, respectively. However, Keri et al. and Evans et al. noted no significant impact on overall success rates or procedure time, respectively. Tsai et al.'s study provides compelling evidence that a phantom PNCL simulation education course can significantly enhance the confidence levels, skill acquisition, and learning satisfaction of participants. Furthermore, the incorporation of the angle reference device, LAGA, was shown to markedly improve procedural outcomes, thus underscoring its potential utility in enhancing PNCL training. However, the lack of improvement in procedure performance after ten simulation rounds suggests a plateau in learning, highlighting the need for more diverse and complex training scenarios, possibly integrating AR for a more comprehensive educational experience. The studies collectively indicate that AR has the potential to enhance IR training, particularly in improving accuracy, confidence, and adherence to procedures. However, its effectiveness varies with the complexity of the procedure, the specific AR technology used, and the experience level of the operators. While AR shows promise in bridging the gap between novice and experienced practitioners, its application in real-world clinical settings and its impact on long-term skill retention remain areas for further exploration.

Our review highlights the contrasting roles of wearable AR devices (e.g., Microsoft HoloLens™, Google Glass™) and AR-enhanced simulators (e.g., Perk Tutor™) in ultrasound-guided procedural training. Wearable AR devices excel in offering immersive experiences by overlaying holographic data onto the user's visual field, improving spatial awareness and procedural precision. This immersive approach has been shown to significantly enhance needle positioning and procedural efficiency. On the other hand, AR-enhanced simulators prioritize procedural skill development, providing interactive feedback and realistic simulations, though their impact on learning outcomes has been more variable. This dichotomy underscores the complementary benefits of wearable AR for spatial and accuracy enhancements, versus simulators for hands-on practice and skill refinement. The integration of both technologies could offer synergistic advantages, suggesting a promising avenue for future research in optimizing IR training.

Offering innovative methods for teaching complex procedures holds the potential for improving IR training. Anderson et al. described an AR simulation system specifically designed for interventional radiology. This system enables real-time manipulation of catheters and guidewires through a tactile user interface, closely mimicking the hand-eye coordination required in actual procedures. It includes additional features like the administration of contrast and C-arm control, offering a comprehensive simulation experience. Feedback from neurological and peripheral vascular interventionalists indicated that while the system captured 75% of the desired functionality and was beneficial for educational simulation, further enhancements were needed for it to be effective in preoperative planning [24]. The capacity for AR to generate immersive scenarios within genuine IR suites offers a dual advantage: familiarisation with real-world settings and practice before tackling complex cases [25]. Additionally, collaborative experiences facilitated by AR promise a broader spectrum of learning. AR enhances the learning experience by providing an interactive, three-dimensional perspective, which is not possible with traditional video recordings, thus offering a more comprehensive and engaging way for learners to understand and practice complex IR procedures [26]. The potential for AR to level the playing field between novices and experts in IR is promising. Studies such as that by Huang et al. are particularly illustrative, revealing how AR aids even untrained individuals in matching the performance metrics of seasoned experts [15]. Utilizing AR recurrently in IR education can enable trainees to proficiently adapt to this technology, potentially leading to substantial advantages in the future, as AR-guided procedures are poised to become standard practice in IR [27].

However, the adoption of AR is not without its considerations. Challenges about AR utilization, such as headaches, dizziness, ergonomic constraints, and initial costs, are real and have been previously noted [28]. Feedback from the results, including Huang et al. mention of tool adjustment issues. Wu et al. note that initial unfamiliarity with devices highlights practical impediments. Moreover, as one delves deeper into the evidence, the unequivocal superiority of AR as an instructional tool is yet to be universally established. Some studies have found negligible differences between AR-enhanced methods versus conventional instruction, highlighting the need for a more nuanced approach to its integration [29]. A salient challenge revolves around the dynamic nature of the human body, organ systems can exhibit respiratory-dependent movement, like the liver and lungs, introducing an element of unpredictability. The precision of AR applications in such scenarios remains a subject of rigorous scrutiny, as static preclinical studies on phantoms might not effectively replicate these real-life complexities. Although concerns are made regarding the initial setup costs of AR technologies there is a potential for AR to provide cost-effectiveness in healthcare education, recent studies have begun to elucidate the balance between AR's initial investment costs and its long-term benefits. Khor et al. suggest the cost-saving potential of AR technologies in surgical training, highlighting the reduction in training time and resources as key factors that could offset the initial setup costs [30].

The embrace of AR in medical education has unearthed a plethora of opportunities for a more interactive, immersive, and efficient training process. Across alternative specialties, the promise of AR in enhancing the learning process is becoming evident for its potential to enhance both procedural and theoretical learning. In thoracic surgery, for instance, Rad et al. illustrated how AR's capacity to vividly showcase intricate anatomies led to a noticeable decrease in preoperative preparation time [31]. A review by Gemini et al. underscored AR's potential as a tool that not only complements traditional pedagogical methods in IR training but also enhances the cognitive and motor skills of learners [8]. This enhancement is particularly salient when considering the nuances of IR, where spatial understanding, intricate hand-eye coordination, and swift decision-making become paramount. Expanding on these insights, Barteit et al. emphasized AR's benefits in repeated practice without patient risks in surgery and anatomy​​, while Sadek et al. documented its efficacy in improving surgical procedure times and trainee confidence during the COVID-19 pandemic​​ [32,33]. 

Expanding on these applications, the potential future trajectory of AR in medical care extends beyond procedural training, promising to transform patient care, diagnosis, and treatment planning. As AR technologies evolve, they offer unique opportunities to enhance patient engagement, surgical accuracy, and remote care delivery. For instance, AR can facilitate more precise surgical planning and execution by overlaying critical anatomical information during procedures [30]. Additionally, its application in robotic-assisted surgery demonstrates significant improvements in procedural outcomes and patient safety [34]. However, the realization of AR's full potential requires addressing current limitations, including technological challenges and the need for robust clinical evidence [30].

This review, while comprehensive, bears certain limitations. The small sample sizes utilized in the studies represented a significant limitation, thereby hindering the ability to generalize the findings broadly. Furthermore, it is essential to note that these studies primarily used phantoms, not actual human subjects. This raises concerns about the direct transferability of findings, as the tactile experience and complexities associated with human patients could differ substantially from phantom models. Additionally, a key limitation to consider is the scarcity of studies focusing on Interventional Radiology trainees, residents, and specialists. Furthermore, the reliance on short-term outcome measures in these studies, rather than long-term skill retention and clinical impact, presents another significant gap in the current research. A more robust approach involving larger, randomized trials with standardized outcome measures is needed for a deeper understanding of AR's efficacy in IR training.

The advent of AR stands as a nascent yet burgeoning frontier, potentially reshaping traditional procedures with its synergy of artificial intelligence, haptics, and other breakthrough technologies [35]. In the spirit of the UK's Innovation Strategy, fostering an international AR-in-IR community could catalyze collaborations, standardization, and resource sharing, propelling the healthcare sector into a new era of innovation and inclusivity. For stakeholders ranging from educators to policymakers and technology developers, the task at hand is unequivocal: to embrace AR's transformative capabilities while addressing its challenges, thereby sculpting the future of interventional radiology in a manner that harmonizes with our national pursuit for innovation. Emphasis should also be placed on longitudinal studies to assess the long-term effectiveness and impact of AR in clinical practice, and on developing user-friendly and cost-effective AR solutions that can be widely adopted, ensuring a harmonized progression in medical education.

## Conclusions

This review underscores the potential of AR in transforming procedural IR education, promising to revitalize learning paradigms and enhance experiential training. While AR emerges as a formidable ally in bridging gaps in visualization and fostering a deeper spatial understanding, it concurrently introduces significant challenges, including implementation costs, adaptability hurdles, and accessibility concerns that cannot be overlooked. As we consider the future landscape of AR within IR, it becomes evident that addressing specific considerations is crucial to fully leverage AR's capabilities and effectively mitigate its limitations. The seamless integration of AR into clinical practice emerges as a primary concern, necessitating research into how AR can be incorporated into clinical workflows to enhance procedural precision and patient outcomes without disrupting established practices. Additionally, the question of skill retention following AR-assisted training calls for immediate investigation. With the rapid pace of technological advancement, it is vital to understand the longevity of skills acquired through AR in the clinical setting, which is essential for validating the long-term benefits of AR in medical education. As the narrative of IR education continues to evolve, adopting a judicious approach to integrating AR is paramount. This approach promises a harmonized and enriched training landscape while carefully navigating potential pitfalls. Prioritizing research on clinical integration, skill retention, standardization, cost-effectiveness, and ethical considerations will be instrumental in advancing AR's application in IR. By addressing these key considerations, we can not only optimize the benefits of AR for interventional radiologists and their patients but also ensure the effective and sustainable integration of AR into IR education and practice.
